# What determines the preference for future living arrangements of middle-aged and older people in urban China?

**DOI:** 10.1371/journal.pone.0180764

**Published:** 2017-07-17

**Authors:** Dijuan Meng, Guihua Xu, Ling He, Min Zhang, Dan Lin

**Affiliations:** 1 Nursing College, Nanjing University of Chinese Medicine, Nanjing, Jiangsu Province, China; 2 Department of General Surgery, Affiliated Hospital of Nanjing University of Chinese Medicine, Jiangsu Province Hospital of TCM, Nanjing, Jiangsu Province, China; 3 Department of Nursing, Medical College of Jiangsu University, Zhenjiang, Jiangsu Province, China; Stanford University, UNITED STATES

## Abstract

**Objective:**

Living arrangements are important to the elderly. However, it is common for elderly parents in urban China to not have a living situation that they consider ideal. An understanding of their preferences assists us in responding to the needs of the elderly as well as in anticipating future long-term care demands. The aim of this study is to provide a clear understanding of preferences for future living arrangements and their associated factors among middle-aged and older people in urban China.

**Methods:**

Data were extracted from the CHARLS 2011–2012 national baseline survey of middle-aged and elderly people. In the 2011 wave of the CHARLS, a total of 17,708 individual participants (10,069 main respondents and 7,638 spouses) were interviewed; 2509 of the main respondents lived in urban areas. In this group, 41 people who were younger than 45 years old and 162 who had missing data in the variable “living arrangement preference” were excluded. Additionally, 42 people were excluded because they chose “other” for the variable “living arrangement preference” (which was a choice with no specific answer). Finally, a total of 2264 participants were included in our study.

**Results:**

The most popular preference for future living arrangements was living close to their children in the same community/neighborhoods, followed by living with adult children. The degree of community handicapped access, number of surviving children, age, marital status, access to community-based elderly care centers and number of years lived in the same community were significantly associated with the preferences for future living arrangements among the respondents.

**Conclusion:**

There is a trend towards preference for living near adult children in urban China. Additionally, age has a positive effect on preference for living close to their children. Considerations should be made in housing design and urban community development plans to fulfill older adults’ expectations. In addition, increasing the accessibility of public facilities in the residential area was important to the elderly, especially for those who preferred living in proximity to their children rather than co-residing with their children. We found that more surviving children were associated with a lower likelihood of choosing “institutionalization”, and it positively contributed to preference for intergenerational living arrangements in our study. As expected, compared with their married counterparts, people who were separated/divorced/widowed preferred living with adult children rather than living independently. A relatively shorter length of residence in the same community was an important indicator of preference for independent living; this finding might require further research.

## Introduction

The world is entering largely unfamiliar territory with respect to population aging. The World Health Organization (WHO) has estimated that the proportion of the world’s older adults aged 60 years or above will nearly double from 12% to 22% between 2015 and 2050 [1]. Similarly, the population is aging rapidly in China because of the lower mortality rate and the one-child policy. The proportion of the older population (≥65 years) in China is expected to increase rapidly from 8.3% to 22.6% between 2010 and 2040 [2].

Unlike in developed countries, where almost all elderly have access to publicly provided social security, the family is the main source of support for Chinese elderly adults [3]. In addition, multigenerational family households had once been the dominant living arrangements for this population [4]. This tradition is attributed to Confucian ideals that required children to obey and serve their parents [5]. However, economic development, urbanization, and other sociological factors have brought fundamental changes to the family structure and living arrangements of the elderly in China. Meanwhile, concomitant socioeconomic changes can affect the attitudes and values surrounding traditional modes of old-age support [6].

Researchers in the United States have shown that the proportion of elderly who live independently has increased dramatically in the twentieth century [7–9]. A similar trend has been noted in China, where co-residence of elderly parents and married children has significantly declined for decades, especially for those in urban areas [10–12]. The trend has raised concerns over the reliability of families to provide support for the elderly in China. Determinants of elderly living arrangements have gained considerable attention from researchers and policy makers to implement effective health policies that are designed to ease strains on state finances and address the health care needs of the elderly. From this perspective, Japan’s public long-term care insurance (LTCI) system was introduced in April 2000, which aims to help elderly people live independently in their homes for as long as possible [13]. Social norms and personal preferences of the older people themselves are the most important influencing factors of living arrangement choices among the elderly [14].

The variables that are correlated with older adults’ preferences for future living arrangements have been measured differently in the literature. Diwan, Lee and Sen found that expectations of filial obligation, the length of residence in the U.S., and self-rated health are significantly associated with living arrangement preferences in Asian Indian immigrants [15]. Silverstein and Angelelli argue that elderly parents who expect to move closer to adult children tend to be older and female and to have at least one child who is better-off than they are; additionally, parents are more likely to expect to move closer to a daughter than to a son [16]. A qualitative study of preferences for future care shows that past experiences, family structure, and current relationships with children and grandchildren influence their perceptions of how they would manage in the future [17]. According to Beland, compared with those living with children, relatives, or friends, older adults living alone or with only a spouse are more likely to prefer to move into an alternate setting (senior housing or nursing home) [18]. A study on the preference for living arrangements among the elderly in India notes that the majority of Indian elderly adults prefer to be in a co-residence, and those who are younger and females and those with no sons prefer to live alone compared with their counterparts [19].

Several studies have been conducted to evaluate the patterns and determinants of current living arrangements of the Chinese elderly and their association with old-age psychological health [3, 20–23]; however, to date, very little information is available on the preferences for living arrangements among the elderly in China’s urban area. Even fewer studies have examined the living arrangement expectations of middle-aged adults. In addition, we consider not only individual characteristics but also social environmental factors that may influence living arrangement preferences in our design. Wister and Burch suggested that extending our understanding of preferences and attitudes in living arrangements will help us respond to the needs of the elderly as well as forecast future demand [24]. Therefore, the purpose of this study is to obtain an understanding of various factors related to the preference for future living arrangements among middle-aged and older people in urban China.

## Materials and methods

### Sample

We obtained data from the China Health and Retirement Longitudinal Survey (CHARLS) 2011–2012 national baseline survey [25–26], which is described in detail by Zhao et al [27]. Samples were chosen through multistage probability sampling. In the first stage, 150 county-level units that fell within 28 provinces were randomly chosen with a probability-proportional-to-size (PPS) sampling technique from a sampling frame containing all county-level units, except Tibet. The sample was stratified by region and within region by urban districts or rural counties and per capita statistics on gross domestic product (GDP). The sample used the lowest level of government organization, consisting of administrative villages (cun) in rural areas and neighborhoods (shequ or juweihui) in urban areas, as primary sampling units (PSUs). Three PSUs were selected within each county-level unit using PPS sampling. In the 2011 wave, a total of 17,708 individual participants responded; 10,069 were main respondents, and 7,639 were spouses of main respondents. Couples rather elderly persons make the decision regarding living arrangement. Therefore, we chose our sample from the main respondent rather than all participants. There are 2509 main respondents who live in urban areas in the CHARLS. Forty-one people who were younger than 45 years old and 162 people with missing data for the variable “living arrangement preference” were excluded. Additionally, 42 people were excluded because they chose “other” for the variable “living arrangement preference” (a choice with no specific answer). Finally, a total of 2264 participants were included in our study.

### Ethical statement

The Statistics Act of China allowed the CHARLS to conduct the nationally representative longitudinal survey of people in China who were 45 years of age or older and their spouses [27]. The first author applied to access the data and obtained approval from the CHARLS survey team to obtain the data. All records and information had originally been anonymized, de-identified and coded. All respondents had signed written consent to participate in the survey when it was conducted by the CHARLS.

### Measurement

Preference for future living arrangement was measured by asking respondents the following question: “If an elderly person has a spouse and adult children and has a good relationship with them, what do you think is the best living arrangement for him/her?” Respondents were asked to select one option from a list of five: a) live with adult children; b) do not live with them but live in the same community or village (live near adult children); c) do not live with them in the same house or the same community or village (live independently); d) live in a nursing home; and e) other.

The factors used to analyze the determinants of living arrangement preferences were constructed as follows. Social and environmental factors included years lived in the same community, the presence of community-based elderly care center, and the degree of community handicapped access. Years lived in the same community were classified into 3 categories: 10 years or less, 11–25 years and more than 25 years. The rationale for assessing years lived in the same community was to examine the role of social networks through neighbors, friends and relatives in preferences for one’s living arrangement [28]. Access to community-based elderly care services was assessed in an attempt to evaluate its impact on living arrangement preferences [29], and it was transformed into a dichotomous variable (presence versus absence). The degree of community handicapped access was assessed using a 7-point Likert Scale with 1 representing “no handicapped access” and 7 indicating “very convenient”. In addition, individual resources consisted of the number of surviving children, availability of children, self-rated health and self-rated standard of living. The number of surviving children (classified into 3 categories: zero, one and two or more) has been extensively used in similar studies [16, 19]. The availability of children, based on a previous study [15], was classified into 3 categories: daughters only, sons only, and daughters and sons. Self-rated health was categorized as poor, fair, good, and very good/excellent. One indicator of wealth information available in the CHARLS survey was self-rated standard of living, which was classified into 3 categories: poor/relatively poor, average, and very high/relatively high. Psychological factors consisted of depression and life satisfaction. In the CHARLS, depressive symptoms were measured with a 10-item version of the Center for Epidemiologic Studies Depression (CES-D10) Scale (≥10 indicating clinical depression). Life satisfaction was examined as a dichotomous variable (satisfied versus not satisfied). The demographics included age, gender, marital status and education. Age was classified into 3 categories: 45–59, 60–74, and over 75 years old. Marital status was categorized as currently married, separated/divorced, and widowed. Education level was divided into four groups that ranged from no schooling to college and above.

### Statistical analysis

Descriptive analyses, such as the frequencies, mean values and standard deviations, were used to examine the sample characteristics. As preference for future living arrangement was a non-ordered categorical variable, a multinomial regression model was performed using SPSS Version 20.0 (IBM SPSS, Armonk, NY, USA) to estimate the associations between various attributes and preferences for future living arrangements. The expected utility of the preference for living with adult children was normalized to zero, and the other three categories of preferences were interpreted in relation to this reference category. Living with adult children had once been the most prevalent living arrangement and the best option for Chinese elderly people [20]. Following a previous study [28], we present the estimated coefficients. The exponential value of the estimated coefficients, however, gives the change in the probability of the alternative (relative to preference for co-residence with adult children) for a unit change in the explanatory variable.

## Results

### Characteristics of the respondents

Table 1 below displays the characteristics of the respondents. The proportion of men and women was approximately equal, and the average age of the respondents was 59.9 (±10.2) years. The oldest respondent among the elderly was 101 years old. Nearly half (49.1%) had an education level of middle school or above. Most had sons (72.2%), fairly good health status (79.5%) and not bad self-rated standard of living (56.1%), did not suffer from depression (77.9%), were satisfied with life (87.6%), lived in the same community for more than 10 years (62.8%) and had no access to community-based elderly care service (73.7%). The average score of the degree of community handicapped access in the community was 2.92 (±1.6), indicating that the community/neighborhoods they lived in were relatively inaccessible. The majority (91.3%) preferred to live with their children or live in the vicinity of their children.

**Table 1:** Characteristics of the sample (N = 2264).

	n	%
**Female**	1313	58.0
**Age in 2011(Mean = 59.9±10.2)**		
45–59	1216	53.7
60–74	809	35.7
≥75	239	10.6
**Marital status**		
Married	1796	79.3
Separated/divorced	98	4.3
Widowed	370	16.3
**Education level**		
No schooling	278	12.3
Primary school and less	658	29.1
Middle school/high school	1112	49.1
College and above	214	9.5
**Number of surviving children**		
0	58	2.6
1	757	33.4
≥2	1449	64.0
**Availability of children**		
Sons only	852	43.9
Daughters and sons	549	28.3
Daughters only	540	27.8
**Self-rated health**		
Poor	463	20.5
Fair	1180	52.2
Good	419	18.5
Very good/excellent	200	8.8
**Self-rated standard of living**		
Poor/relatively poor	978	43.9
Average	1163	52.2
Very high/relatively high	87	3.9
**Depression**		
Depressed	482	22.1
Not depressed	1696	77.9
**Life satisfaction**		
Satisfied	1842	87.6
Not satisfied	260	12.4
**Access to community-based elderly care service**		
Yes	563	26.3
No	1580	73.7
**Years lived in the same community**		
≤10	532	37.2
11–25	513	35.9
>25	385	26.9
**Preferred future living arrangement**		
Live with children	932	41.2
Live in the vicinity of children	1135	50.1
Live independently	118	5.2
Live in a nursing home	79	3.5

### Percentage distribution of respondents’ preferred living arrangements by their characteristics

Table 2 presents the results of the R×C table Chi-square test for the percentage distribution of respondents’ preferred living arrangements by their characteristics. The respondent characteristics that were significantly associated with their preference for future living arrangements were age (p = 0.001), education level (<0.001), availability of children (p = 0.008), self-rated standard of living (p = 0.042) and years lived in the same community (p = 0.002).

**Table 2:** Percentage distribution of respondents’ preferred living arrangements by their characteristics.

	Live with adult children n (%)	Live in vicinity of children n (%)	Live independ-ently n (%)	Live in a nursing home n (%)	P value
**Gender**					
Male	385 (40.5)	475(49.9)	62(6.5)	29(3.0)	0.090
Female	547(41.7)	660(50.3)	56(4.3)	50(3.8)	
**Age in 2011(Mean = 58.1±9.2)**					
45–59	541(44.5)	578(47.5)	51(4.2)	46(3.8)	0.001
60–74	309(38.2)	419(51.8)	51(6.3)	30(3.7)	
≥75	82(34.3)	138(57.7)	16(6.7)	3(1.3)	
**Marital status**					
Married	716(39.9)	919(51.2)	97(5.4)	64(3.6)	0.104
Separated/divorced/widowed	216(46.2)	216(46.2)	21(4.5)	15(3.2)	
**Education level**					
No schooling	135(48.6)	120(43.2)	16(5.8)	7(2.5)	<0.001
Primary school and less	307(46.7)	288(43.8)	39(5.9)	24(3.6)	
Middle school/high school /college and above	488(36.8)	727(54.8)	63(4.8)	48(3.6)	
**Number of surviving children**					
0	19(32.8)	32(55.2)	4(6.9)	3(5.2)	0.227
1	306(40.4)	392(51.8)	29(3.8)	30(4.0)	
≥2	607(41.9)	711(49.1)	85(5.9)	46(3.2)	
**Availability of children**					
Sons only	401(47.1)	397(46.6)	34(4.0)	20(2.3)	0.008
Daughters and sons	231(42.1)	268(48.8)	35(6.4)	15(2.7)	
Daughters only	213(39.4)	272(50.4)	28(5.2)	27(5.0)	
**Self-rated health**					
Poor	190(41.0)	227(49.0)	25(5.4)	21(4.5)	0.079
Fair	504(42.7)	568(48.1)	70(5.9)	38(3.2)	
Good/very good/excellent	237(38.3)	339(54.8)	23(3.7)	20(3.2)	
**Self-rated standard of living**					
Poor/relatively poor	415(42.4)	465(47.5)	54(5.5)	44(4.5)	0.042
Average/very high/relatively high	500(40.0)	654(52.3)	61(4.9)	35(2.8)	
**Depression**					
Depressed	206(42.7)	234(48.5)	24(5.0)	18(3.7)	0.722
Not depressed	682(40.2)	871(51.4)	86(5.1)	57(3.4)	
**Life satisfaction**					
Satisfied	757(41.1)	938(50.9)	90(4.9)	57(3.1)	0.052
Not satisfied	107(41.2)	121(46.5)	16(6.2)	16(6.2)	
**Access to community-based elderly care service**					
Yes	249(44.2)	270(48.0)	26(4.6)	18(3.2)	0.164
No	616(39.0)	815(51.6)	89(5.6)	60(3.8)	
**Years lived in the same community**					
≤10	212(39.8)	260(48.9)	43(8.1)	17(3.2)	0.002
11–25	175(34.1)	299(58.3)	24(4.7)	15(2.9)	
>25	167(43.4)	185(48.1)	16(4.2)	17(4.4)	

Using the Bonferroni correction, we performed multiple comparisons of respondents’ preferred living arrangements between different age groups, and statistical significance was attained in comparisons of each group. Surprisingly, the proportion of preference for co-residence with their children and institutional care was lower among respondents aged 75 years old and above, while the proportion was higher among those who preferred to live in the proximity of their adult children.

As with age, our analysis showed differences by education level (no schooling vs middle school and above, primary school vs middle school and above) in living arrangement preferences. The proportion of respondents who preferred to live with their adult children was inversely related to the level of education that they had attained. The proportion preferring to live close to their children was higher among those who were well-educated. In addition, a relatively lower proportion of the uneducated respondents, compared to those who were well-educated, preferred institutional care.

Multiple comparisons analysis showed that the percentage distribution of respondents’ preferred living arrangements differed between those who had daughters only and those who had sons only. The proportion of respondents who preferred to live with their children was 38.2% among those who had daughters only, while the corresponding proportion was 44.9% among those with sons only. Furthermore, a relatively higher proportion of respondents with no sons (4.8%) preferred institutional care compared to those who had at least one son (2.2%).

The analysis showed a clear link between the financial status of the elderly and their living arrangement preferences. A relatively higher proportion of the respondents (52.3%) who reported not bad living standards preferred to live near their children compared to those whose self-rated standard of living was poor/relatively poor (47.5%). The proportion of respondents who preferred to live in a nursing home was higher among those with poor/relatively poor self-rated standard of living compared to those with a not bad self-rated standard of living.

Our analyses showed that the percentage distribution of respondents’ preferred living arrangements by different lengths of time lived in the same community were all different. A relatively higher proportion of the respondents (43.4%) who reported living in the same community for more than 25 years preferred to live with their children compared to those who lived in the same community for 11–25 years (34.1%). Similarly, a higher proportion of respondents among those who lived in the same community for 11–25 years (58.3%) preferred to live in the proximity of their children. Furthermore, the proportion preferring to live independently (8.1%) was higher in respondents who lived in the same community less than or equal to 10 years.

### Factors associated with the preference for future living arrangements

Table 3 presents the results of the multinomial regression analysis for three categories of preferences compared to “live with adult children”, which was the reference category. The overall model was significant (Wald chi square = 111.731, p<0.05).

**Table 3:** Multiple logistic regression analysis of respondents’ characteristics and living arrangement preferences.

	Live in vicinity of children coeff.	Live independently coeff.	Nursing home coeff.
**Female**	0.165	-0.344	0.676
**Age (in 2011)**	0.036*	0.032	0.046
**the degree of community handicap access**	0.097*	0.126	-0.252
**Number of surviving children**	-0.261*	-0.050	-0.648*
**Marital status:** married(ref.)			
Separated/divorced/ widowed	-0.358	-0.945*	0.075
**Completed education:** no schooling(ref.)			
Primary school and less	0.470	-0.407	0.588
Middle school/high school/college and above	-0.006	-0.352	0.558
**Availability of children:** daughters only (ref.)			
Sons only	-0.177	-0.394	-0.642
Daughters and sons	0.166	0.313	-0.675
**Self-rated health**: good/very good/excellent(ref.)			
Poor	-0.255	0.081	0.095
Fair	-0.238	0.345	0.134
**Self-rated standard of living:** average /very high/relatively high(ref.)			
Poor/relatively poor	0.007	0.258	0.008
**Depression**: depressed (ref.)			
Not depressed	-0.040	0.003	0.991
**Life satisfaction:** not satisfied(ref.)			
Satisfied	0.120	-0.128	-0.642
**Access to community-based elderly care service:** no (ref.)			
Yes	-0.125	-0.932*	0.056
**Years lived in the same community:** longer than 25years (ref.)			
≤10	0.177	0.897*	-0.009
11–25	0.336	0.418	-0.119
**constant**	-2.017	-3.958	-4.297
**observation**	2264		
**-2Log-likelihood**(only intercept)	2054.288		
**-2Log-likelihood**(final)	1942.557		

* Significant at 0.05.

#### Preference for living in the vicinity of children

The factors that were significantly associated with preference for living in the vicinity of children among the respondents in multiple logistic regression analysis were age, degree of community handicapped access, and number of surviving children. Increased age and convenient community handicapped access were significantly associated with increased odds of preference for living in the vicinity of children compared to preference for living with adult children. Those with more surviving children were more likely to choose “living with their adult children” than “living in the vicinity of their children”.

#### Preference for living independently

Living in the same community for less than or equal to 10 years was significantly associated with increased odds of preference for living independently compared to preference for living with adult children. For those who preferred living with adult children to living independently, the significant predictors were marital status and access to community-based elderly care service. People who were separated/divorced/widowed were less likely to choose “living independently” compared to “living with adult children”. The same findings were observed for people who had access to a community-based elderly care center, which was unexpected.

#### Preference for living in a nursing home

The final model showed that for predicting the choice of living in a nursing home, the only variable that approached significance was the number of surviving children. The number of surviving children was significantly associated with decreased odds of preference for living in a nursing home in the future. People with more surviving children were less likely to choose “living in a nursing home” in the future compared to “living with adult children".

## Discussion

In our study, the most preferred choice for future living arrangements was to live near children. This finding was consistent with those of previous studies. A study among middle-aged and older Asian Indian immigrants reported that the most popular preference for future living arrangements was to “move closer to children” [15]. Bian et al. [30] found a similar co-residence pattern “proximity to parents” in urban China. Lei et al. [3] argued that a large fraction of Chinese elderly who live alone or with a spouse only have an adult child living nearby to provide care when needed, which implies that living close to children has become a prevalent approach to providing old-age support while maintaining the independence/privacy of both elders and their adult children.

This present study showed that 41.2% of the participants preferred to reside with adult children, which agreed with the situation reported by Lei et al [3] that approximately 41% of Chinese aged 60 years and over currently live with an adult child. The proportion of respondents who expressed preferences for living with adult children or living close to children was relatively high (91.3%), which may suggest that most of the Chinese elderly are more likely to expect informal support from family when assistance is needed.

Age, education level, availability of children, self-rated standard of living and years lived in the same community were significantly associated with the preference for future living arrangements among the respondents based on the Chi-square test. To some extent, this analysis revealed useful information, but a Chi-square test is a single factor analysis, akin to correlation, and thus cannot be used to examine the determinants of and predict the likelihood of living arrangement preferences. Therefore, we used a random parameters logit model to identify the determinants of the stated preference for living arrangements among middle-aged and elderly participants.

We found the effect of community handicapped access was positive on the preference for living close to their children in relation to co-residing with children. Environment plays an important role in how well people adjust to loss of function and other forms of adversity that may be experienced in later years [31]. Obviously, a community that has very convenient handicapped access could better meets the needs of their old residents, especially for those who are disabled. Therefore, the possibility remained that people who lived in an inclusive and accessible community might have greater confidence in living separately from their children while keeping close contact with children. Our findings showed people who had access to community-based elderly care service preferred to live with adult children rather than live independently, which was unexplained. However, our analysis could not pinpoint any causal relationships because the dependent variable was a hypothetical response to a question about preferred living arrangements when elderly, while the explanatory variables were the current characteristics of the respondent and their community, and some of the respondents were not yet elderly.

This study indicated the number of surviving children was a significant predictor of living arrangement preference. Compared to living closer to children or entering a nursing home, respondents who had more surviving children were more likely to choose “living with their children”. One possible reason for this finding is that having more children increases the chance that there would be at least one suitable child with whom respondents might prefer to live. This suggests that, under ideal conditions, living with adult children is still a favorite choice, and filial norms still impact the living arrangement of the elderly in China due to its historical and cultural heritage. Panigrahi AK [19] also showed similar results that the proportion of the elderly who preferred to co-reside with children increased with the number of surviving children they had.

In our analysis, the effect of age on the preference for living closer to children compared to living with adult children was positive. In a study of 734 elderly, Hongkonger reported a similar pattern and speculated that younger respondents might prefer co-residence because the social norm was to live with unmarried children [32]. Wong et al [33] reported that Chinese and Korean immigrant elders seemed to be more sensitive to becoming a burden on their children’s families; therefore, these elders modified their expectations by living independently while remaining in close contact with their children. Additionally, it reflected “pure preference”, regardless of the actual situation they faced (e.g., whether they were capable of self-care).

Unexpectedly, compared to those who lived in the same community longer than 25 years, we found respondents who had lived in the same community for a shorter period (≤10 years) were more likely to choose “living independently”. This finding was inconsistent with research conducted in Canada by Sarma [28], who reported that living longer in the same community increased the probability of living independently due to informal home care provided by neighbors, friends and relatives in the community. We could interpret our result from another point of view and consider that those who lived in the same community longer than 25 years were more likely to be defenders of traditional cultural norms and thus preferred to live with their children. However, those who lived in the same community for a shorter time might be prone to adapting to the value system associated with the west and prefer to live independently.

In this study, we found that being married reduced the likelihood to choose “living with adult children” compared to “living independently”. This result was consistent with the actual living arrangements of the oldest old in China [20]. The role of marital status on intergenerational living arrangements has similar findings in the literature [19, 32, 34], which suggests that living without a spouse might be an important predictor of co-residence with adult children. However, the negative effect of marital status on living in a nursing home, which was generally found in the previous studies [28, 34–36], was not supported in our study.

The major limitation of the present study was that the measurement of the living arrangement preference assumed that an elderly person had a spouse and adult children as well as a good relationship with them. Bias may occur because marital status, number of children, parent-child relationships, and relationships between the husband and wife could be factors affecting their preferences for living arrangement. In addition, the explanatory variables we chose may not be the perfect indicators of living arrangement preference because the CHARLS was not specifically designed for our research.

## Conclusions

Despite the fact that our analysis was based on the assumption that respondents had a good relationship with their children, the findings of this study still showed a trend towards preference for living in the vicinity of adult children in urban China. In addition, regardless of the actual options available or cost, even for older people, participants preferred to live close to their children rather than co-reside with children. This finding has implications for housing design and urban community development planning. As suggested by lai [37], considerations have to be made to ensure that provisions for the aging population to live independently in the same or nearby community are available to fulfill their expectations. Guan J et al [38] found that compared to elderly adults with concordant living arrangements, it was much more difficult for elderly adults with discordant arrangements to enjoy their actual living arrangements.

In addition to providing the aging population with different housing choices that allow them to make an ideal living arrangement, the application of a non-barrier design in the residential area is also important for the elderly, which may enable them to choose to live separately from their children in the community.

The negative effect of marital status on intergenerational living arrangements agreed with existing research, while the positive effect of years lived in the same community on preferences for living with adult children found in this study was inconsistent with previous research. Further research is needed to explore the association between the length of residence in the same community and living arrangement preferences in different regional and cultural backgrounds.

Having more surviving children increased the likelihood of expecting to co-reside with their children, and it will be necessary for future research to consider the characteristics and resources of the children, such as their education level and household income, to better understand the determinants of elderly living arrangement preferences. Longitudinal data on living arrangements are also needed to develop a clearer picture of the link between preferences and actual arrangements.
